# Frontier research and clinical application prospects of microbiome biomarkers in autoimmune diseases

**DOI:** 10.3389/fimmu.2026.1752840

**Published:** 2026-07-17

**Authors:** Qianqian He, Pinjun Zhang, Zhenni Chen, Chengping Wen, Mingzhu Wang

**Affiliations:** Research Institute of Chinese Medical Clinical Foundation and Immunology, School of Basic Medical Science, Zhejiang Chinese Medical University, Hangzhou, China

**Keywords:** autoimmune diseases, biomarkers, clinical translation, microbiome, precision medicine

## Abstract

The microbiome is increasingly recognized as a master regulator of immune homeostasis and a key environmental factor associated with the pathogenesis of autoimmune diseases (ADs). This review comprehensively synthesizes current knowledge on how microbial communities and their metabolites may contribute to ADs’ development through microbial-immune interactions, dysbiosis, and the involvement of viral and fungal components within an integrated inter-kingdom ecosystem. We propose an operational definition of microbiome biomarkers as measurable microbiome-associated features reflecting disease susceptibility, activity, prognosis, or therapeutic response and categorize them into three classes: taxonomic, functional/metabolic, and host–microbiome interaction-derived biomarkers. We critically evaluate the evidence for specific microbial signatures as biomarkers for early diagnosis, disease monitoring, and prediction of therapeutic responses, incorporating evidence grading that distinguishes validated biomarkers from those that remain exploratory and discussing shared versus disease-specific signatures across ADs. The translational potential of microbiome-targeted interventions, including probiotics, prebiotics, and fecal microbiota transplantation, is examined within a personalized medicine framework, with barriers to clinical implementation explicitly addressed. Key confounding factors such as diet, geographic origin, and medication use are highlighted as critical variables shaping microbiome signatures independently of disease. Looking forward, the convergence of multi-omics technologies and artificial intelligence for biomarker discovery, multi-omics integration, and clinical validation promises to unravel the complex microbiome-immune crosstalk, enabling more accurate diagnosis, prognostic stratification, and ultimately, individualized microbiota-informed therapy.

## 1 Introduction

Autoimmune diseases (ADs) represent a spectrum of heterogeneous immune disorders characterized by dysregulated activation of the immune system, leading to aberrant immune responses targeting self-antigens across organs, tissues, and cells. Their pathogenesis encompasses multifactorial mechanisms involving genetic predisposition, environmental triggers, and immune regulatory dysfunction (1–4). Over 80 clinically recognized ADs include rheumatoid arthritis (RA), systemic lupus erythematosus (SLE), Sjögren syndrome (SS), multiple sclerosis (MS), ankylosing spondylitis (AS), inflammatory bowel disease (IBD), and type 1 diabetes mellitus (T1D). ADs afflict over 10% of the global population, with women disproportionately affected (5, 6). Early stage ADs notoriously present with vague symptoms like persistent fatigue, arthralgia, and low-grade fever, manifestations that often mimic common illnesses, leading to delayed diagnoses (7). Contemporary diagnosis remains anchored in canonical immunological markers, particularly through precise measurements of autoantibody serology and comprehensive assessments of cytokine profiles. However, these methods are constrained by their diagnostic accuracy, particularly in achieving optimal sensitivity and specificity. For instance, older adults demonstrate autoantibody positivity without meeting diagnostic criteria for specific ADs. Therapeutic strategies relying on broad-spectrum immunosuppressants exhibit heterogeneous therapeutic responses and dose-limiting adverse effects. These limitations underscore the critical need for novel biomarkers with improved diagnostic specificity and predictive value in treatment response. Advancements in multi-omics profiling and machine learning algorithms offer promising avenues to refine epidemiological surveillance and precision medicine approaches in AD management. These include feature selection methods for biomarker discovery, multi-omics integration frameworks that link microbial taxa to host immune readouts, and predictive models for disease risk stratification. However, the clinical translation of these computational tools requires rigorous external validation and attention to model interpretability to avoid overfitting and ensure real-world applicability.

The microbiome is a general term for the collection of microbial communities and their genomes in a given ecological niche, encompassing bacteria, fungi, viruses, and archaea. These microorganisms form a highly complex symbiotic system under the interplay of biotic and abiotic factors inside and outside the body and play a key role in the maintenance of host health homeostasis and are associated with disease onset and progression (8). Recent studies have confirmed that specific microbial infections may represent potential pathogenic triggers of ADs, and their compositional and functional alterations can influence immunomodulatory pathways. Increasing evidence suggests that microbiome alterations are associated with AD pathogenesis and may provide clinically useful biomarkers for disease monitoring, treatment response prediction, and early disease risk prediction warning (9, 10). However, a standardized definition of “microbiome biomarkers” is still lacking. In this review, microbiome biomarkers are defined as measurable microbial or microbiome-associated features that reflect disease susceptibility, activity, prognosis, or therapeutic response in ADs. Based on their biological origin, these biomarkers can be classified into three categories: (i) taxonomic biomarkers, referring to disease-associated shifts in microbial composition at the phylum, genus, or species level (11); (ii) functional/metabolic biomarkers, referring to microbial genes, pathways, enzymes, and metabolites (12); and (iii) host–microbiome interaction-derived biomarkers, referring to host immune signatures induced by microbial components or dysbiosis (13). From an immunological perspective, microbiome biomarkers are not merely descriptive indicators of dysbiosis. They may reflect innate immune recognition of microbial-associated molecular patterns (MAMPs) by host pattern-recognition receptors (PRRs), as well as damage-associated molecular patterns (DAMPs) released during epithelial stress or barrier disruption (14, 15). In addition, molecular mimicry, bystander activation, and systemic microbial translocation provide plausible mechanistic links between dysbiosis and autoimmunity (16). Mechanistically, the microbiome modulates both innate and adaptive immunity through multiple layers of regulation. PRR signaling, including Toll-like receptors (TLRs), NOD-like receptors (NLRs), and C-type lectin receptors (CLRs), shapes antigen-presenting cell activation, cytokine production, and T-cell polarization (17). In parallel, microbial metabolites such as short-chain fatty acids (SCFAs), bile acid derivatives, and tryptophan catabolites modulate regulatory T-cell differentiation, epithelial integrity, and inflammatory set points. These mechanisms highlight the value of microbiome biomarkers as mechanistically informative indicators rather than purely correlative signatures (18, 19).

Growing research into the microbiome and ADs has gradually revealed the potential utility of microbiome biomarkers for disease surveillance, treatment response prediction, and early disease detection. In terms of disease monitoring, the structure of the gut microbiota shows a dynamic correlation with the activity of ADs. *Prevotella copri* is significantly enriched in untreated patients with newly-onset RA with high disease activity, and its abundance tends to decrease in treated patients with reduced disease activity (20). Longitudinal studies show that the gut microbiota α-diversity index exhibits a marked rebound in patients entering remission, suggesting that microbiome dynamics can serve as a valuable bioindicator for monitoring disease progression. *Ruminococcus gnavus* has been associated with anti-double-stranded DNA (dsDNA) antibody production in SLE patients through molecular mimicry, and *Ruminococcus gnavus* abundance is positively correlated with SLEDAI score (21). In terms of treatment response prediction, the bidirectional modulatory effects of immunomodulators on the host microbiome provide new directions for precision medicine. Methotrexate treatment responders show a significant reduction in the abundance of *Bacteroidetes* in the gut, suggesting that specific flora characteristics may serve as biomarkers for drug response prediction (22). In terms of early disease risk prediction, altered microbiome α-diversity can be detected at the preclinical stage of certain ADs. For example, reduced gut *Bifidobacterium* abundance precedes T1D onset, accompanied by the expansion of opportunistic pathogenic bacteria (23). Animal experiments have been conducted to further demonstrate that colonization by specific microbial communities significantly modulates the autoimmune response. For example, *segmented filamentous bacteria* (SFB) colonization can promote autoimmune processes through Th17 cell differentiation (24). While current evidence supporting early interventions remains sparse, integrating multi-omics approaches, from macrogenomics and metabolomics to single-cell sequencing, promises to systematically map microbiome-immunity interaction networks and offer critical insights for early stage ADs prevention and targeted control strategies.

Emerging developments in microbiome research have unveiled novel insights into ADs, spanning from predictive modeling to therapeutic interventions. Integrated multi-omics technologies are rapidly advancing the clinical translation of microbial biomarkers, paving the way for novel microbiota-based interventions, including probiotic development, fecal transplants, and targeted metabolite modulation. These advancements not only bridge mechanistic exploration and clinical application but also pioneer individualized treatment paradigms for the management of ADs.

## 2 The association mechanism between the microbiome and ADs

### 2.1 Microbial-immune interaction

Multiple core microbial-immune interaction pathways driving autoimmunity are summarized in **Table 1**, as detailed below. The microbiome influences autoimmune pathogenesis through complex crosstalk with the host immune system. At the interface between commensal microorganisms and host mucosa, microbial products may be sensed by PRRs expressed on epithelial cells, macrophages, dendritic cells, and neutrophils (25, 26). These receptors include TLRs, NLRs, and CLRs, which recognize MAMPs such as lipopolysaccharide, peptidoglycan, flagellin, and fungal β-glucans (27). Activation of these signaling pathways induces the production of inflammatory cytokines, chemokines, and type I interferons, thereby shaping the balance between immune tolerance and autoimmunity (28).

**Table 1:** Multidimensional regulatory mechanisms of microbe-immune interactions.

Mechanism	Mechanism of action and immune effects	Key microorganisms /components	References
Molecular mimicry	Microbial antigenic epitopes can exhibit molecular mimicry with host antigens. Activation of the TLR4/MyD88 signaling pathway by microbial-derived signals triggers the activation of cross-reactive T cells, which infiltrate host tissues and thereby initiate systemic autoimmune responses.	*Enterococcus gallinarum*	(90)
Microbial metabolite dysregulation	The reduction in SCFAs contributes to impaired Treg differentiation and Th17 overactivation resulting from secondary bile acid deficiency. These synergistic effects shift the Th17/Treg balance toward a pro-inflammatory phenotype, ultimately disrupting immune tolerance mechanisms.	*Faecalibacterium prausnitzii*	(91)
Intestinal barrier impairment	The reduced secretion of mucin-degrading enzymes, combined with the downregulation of tight junction protein expression, like ZO-1 and occludin, compromises intestinal barrier integrity; this impairment subsequently initiates systemic inflammatory responses.	*Akkermansia muciniphila*; *Bacteroides*	(92)
Th17/Treg imbalance	Microbiota contribute to the pathogenesis of RA and IBD through the activation of specific Th cell subsets. Notably, Th17 cell activation promotes local IL-17A secretion, which exacerbates intestinal inflammation and systemic immune dysregulation.	SFB	(93)
ILC polarization	Antigen-presenting cells secrete IL-12 in response to intestinal microorganisms, which induces the downregulation of RORγt and upregulation of T-bet in group 3 innate lymphoid cells (ILC3s). This transcriptional reprogramming drives the conversion of ILC3s to ILC1, consequently reducing IL-22 production at inflammatory sites. The diminished IL-22 levels impair mucosal barrier repair, thereby exacerbating inflammatory responses through JAK-STAT signaling pathway activation.	SCFAs	(94, 95)
Epitope spreading	Persistent microbial stimulation induces T cells to recognize novel epitopes, thereby driving the immune system to amplify autoantibody-mediated responses. This cascade exacerbates MS through enhanced immune activation, likely mediated by mechanisms involving epitope spreading and dysregulated adaptive immunity.	*Prevotella copri*	(96)

SCFAs, short-chain fatty acids; Th17, T helper 17 cells; Treg, regulatory T cells; ILC, innate lymphoid cells; TLR4, Toll-like receptor 4; MyD88, myeloid differentiation primary response 88.

In genetically susceptible hosts, persistent microbial stimulation may contribute to breaking immune tolerance through molecular mimicry, epitope spreading, and bystander activation (16). Molecular mimicry refers to cross-reactivity between microbial and self-antigens, as exemplified by *Ruminococcus gnavus*-associated anti-dsDNA responses in SLE (21, 29). Bystander activation may occur when inflammatory cytokines elicited by infection or gut dysbiosis activate autoreactive lymphocytes in an antigen-independent manner. Together, these mechanisms provide a mechanistic basis for the transition from dysbiosis to chronic autoimmunity (30).

At the signaling level, these diverse microbial triggers converge on a limited set of intracellular pathways that collectively drive autoimmune inflammation. Activation of TLRs and NLRs by MAMPs and DAMPs engages the MyD88-dependent cascade, leading to nuclear translocation of NF-κB and the transcription of pro-inflammatory cytokines such as IL-6, TNF-α, and IL-1β (31). Simultaneously, fungal β-glucan recognition via Dectin-1 signals through Syk/CARD9 to promote Th17 polarization and IL-23 production, reinforcing the inflammatory milieu (32). Barrier disruption further amplifies these pathways. The release of DAMPs from damaged epithelial cells activates NLRP3 inflammasomes, driving IL-1β maturation, while impaired tight junctions facilitate sustained microbial translocation and tonic PRR stimulation (33).

These converging signals ultimately lower the threshold for autoreactive lymphocyte activation, providing a mechanistic explanation for how dysbiosis can transition from a correlative observation to a functional contributor to autoimmune pathogenesis.

### 2.2 Dysbiosis

Dysbiosis denotes substantial structural and functional perturbations in microbial communities that are strongly correlated with disease pathogenesis. Emerging evidence indicates that dysbiosis is strongly correlated with disease pathogenesis, although whether it represents a primary driver, a secondary consequence, or a perpetuating factor remains an active area of investigation. Importantly, dysbiosis is a multidimensional state that cannot be simplified to mere loss of microbial diversity. It encompasses at least three non-mutually exclusive patterns: (i) loss of α-diversity, reflecting the collapse of ecological complexity; (ii) depletion of beneficial symbionts, such as short-chain fatty acid-producing bacteria; and (iii) expansion of pathobionts, which may directly promote inflammation and epithelial injury (34, 35). This dysregulation can compromise intestinal barrier integrity, facilitating the translocation of luminal antigens into systemic circulation, a phenomenon commonly referred to as “leaky gut,” which subsequently contributes to immune hyperactivation and pathogenic autoimmunity (36, 37). At the mucosal level, barrier dysfunction involves not only the epithelial cell layer but also the mucus layer, antimicrobial peptides, and tight junction complexes (38). Proteins such as occludin, claudins, and zonulin regulate paracellular permeability. Zonulin, in particular, is recognized as the endogenous modulator of tight junction disassembly, and its upregulation by microbial signals or inflammatory cytokines can reversibly increase intestinal permeability. Dysregulation of these tight junction proteins can promote microbial translocation and systemic immune activation (39, 40). Therefore, the “leaky gut” concept should be interpreted as a consequence of altered barrier biology rather than a standalone mechanism.

Dysbiosis can be further classified into taxonomic dysbiosis and functional dysbiosis. Taxonomic dysbiosis refers to altered microbial composition, whereas functional dysbiosis refers to changes in microbial gene expression, metabolic output, and host–microbe interactive functions. From an immunological standpoint, functional dysbiosis may be more relevant because microbial metabolites such as SCFAs, bile acids, and tryptophan-derived indoles directly regulate epithelial integrity, T-cell differentiation, and inflammatory tone (41, 42). Such metabolite-driven impairment of the epithelial barrier further exacerbates microbial translocation and systemic immune activation, forming a self-reinforcing cycle that contributes to autoimmunity. In addition, extraintestinal dysbiosis has also been documented in the oral cavity, skin, and respiratory tract and may be relevant in disease-specific contexts such as RA, SS, SLE, and MS (43–45). This supports a compartment-specific view of autoimmune dysbiosis, in which the affected microbial niche may vary by disease phenotype (46).

**Table 2** provides a systematic summary of disease-specific microbial signatures and their mechanistic associations across various ADs, underscoring the pivotal involvement of dysbiosis in autoimmune pathogenesis. While the evidence for gut dysbiosis in IBD, RA, and SLE is relatively robust, data for other ADs such as autoimmune liver disease, Graves’ disease, and psoriasis remain more limited and often derive from single-cohort, cross-sectional studies. Nonetheless, these initial observations warrant further investigation to determine whether dysbiotic patterns in these less-studied diseases converge on common immunomodulatory pathways or represent distinct, disease-specific signatures. This uneven depth of investigation across ADs represents a limitation across the field that must be considered when interpreting the apparent disease-specificity of microbial dysbiosis.

**Table 2:** Disease-specific microbial dysbiosis, functional alterations, and mechanistic links in ADs.

Diseases	Characteristic microbial changes	Key functional/mechanistic alterations	References
Rheumatoid arthritis	*Prevotella copri* ↑; *Faecalibacterium prausnitzii* ↓	reduced SCFA synthesis; impaired Treg differentiation and enhanced Th17 polarization; gut-joint axis and disruption of immune homeostasis.	(97–101)
Systemic lupus erythematosus	*Ruminococcus gnavus* ↑	intestinal barrier damage and increased permeability; *Ruminococcus gnavus* induces anti-dsDNA antibodies via molecular mimicry; systemic microbial translocation.	(21, 102–104)
Sjögren’s syndrome	*Faecalibacterium* ↓a *Streptococcu*s ↑t *Prevotella/Veillonella/Firmicutes* ↑	oral dysbiosis; oral-gut bacterial translocation; activation of the LPS/TLR immune pathway; molecular mimicry and autoimmune amplification; impaired salivary gland mucosal defense.	(46, 72, 105–108)
Multiple sclerosis	*Akkermansia muciniphila* ↑ *Faecalibacterium prausnitzii* ↓ *Butyricicoccus* ↓	mucus layer degradation and increased intestinal permeability; reduced butyrate and impaired anti-inflammatory signaling; altered bile acid metabolism.	(109–111)
Inflammatory bowel disease	*Adherent-invasive Escherichia coli* (AIEC) ↑ *Clostridium* spp. ↓ *Roseburia* ↓ *Desulfovibrio* ↑	reduced SCFA synthesis; impaired tight junction proteins (occludin, claudins, zonulin); hydrogen sulfide-induced mucosal injury.	(112–115)
Ankylosing spondylitis	*Klebsiella pneumoniae* ↑ *Bacteroides fragilis* ↓	molecular mimicry with HLA-B27; impaired immune regulation; increased systemic inflammation; reduced commensal-mediated immune tolerance.	(116–118)
Autoimmune liver disease	*Akkermansia muciniphila* ↓ *Clostridium* ↑ *Veillonella* ↑	*Akkermansia muciniphila* reduction leads to damage of the mucus layer and increased intestinal permeability; *Clostridium* promotes the release of pro-inflammatory factors; an increase in *Veillonella* leads to increased lactate synthesis, causing liver damage.	(119–121)
Graves’ disease	*Bacteroides* ↑ *Faecalibacterium* ↓	*Bacteroides* enrichment is associated with elevated thyroid antibody levels; dysbiosis may trigger autoimmune responses through molecular mimicry mechanisms.	(122)
Type 1 diabetes mellitus	*Bacteroides* ↑ *Lactobacillus* ↓ *Butyrate-producers* ↓	*Bacteroides* activate pro-inflammatory pathways, leading to increased inflammation; a reduction in *Lactobacillus* causes an imbalance in immune tolerance; a decrease in *butyrate producers* affects the protective function of pancreatic β cells.	(89)
Psoriasis	*Prevotella* ↑ *Staphylococcus aureus* ↑ *Malassezia* ↑	*Prevotella* activates the IL-23/IL-17 signaling axis, leading to an inflammatory response; *Staphylococcus aureus* secretes superantigens that exacerbate inflammation; *Malassezia* increases the triggering of Th17 responses, disrupting skin immune responses.	(123, 124)

↑, indicates an increase in abundance; ↓, indicates a decrease in abundance.

### 2.3 The role of viruses and fungi

While bacteria remain the most extensively studied microbial kingdom in ADs, the mycobiome and virome are no longer considered secondary components but integral regulators of immune homeostasis and autoimmunity. Moving beyond isolated descriptions of individual taxa, the microbiome must now be conceptualized as a dynamic, inter-kingdom ecological network, where bidirectional, synergistic interactions between bacteria, fungi, and viruses collectively shape immune responses in the pathogenesis of ADs. For instance, fungal-bacterial metabolic cross-feeding can alter the availability of immunomodulatory metabolites such as SCFAs, while phage-driven lysis of bacterial populations can release MAMPs locally and activate PRR signaling in the gut mucosa (47, 48).

The innate immune system’s recognition of fungal components, primarily mediated by C-type lectin receptors with Dectin-1 playing a pivotal role in recognizing fungal β-glucan MAMPs, constitutes a core feature of this inter-kingdom network (49). It specifically binds to β-glucans, key fungal cell wall components, and triggers downstream inflammatory signaling cascades, which in turn regulate immune cell polarization and cytokine release (50). Mycobiome dysbiosis disrupts this recognition balance, skewing immunity toward Th17 polarization, increasing epithelial inflammation, and breaking immune tolerance, thus directly contributing to the initiation and progression of autoimmunity (48, 51). The virome, particularly bacteriophages, functions as a critical indirect regulator of bacterial dysbiosis, thereby exerting profound effects on ADs (52). Bacteriophages modulate the composition and function of bacterial communities by reshaping their structure, regulating horizontal gene transfer between bacterial strains, and altering bacterial virulence or metabolic outputs, which indirectly influence immune homeostasis and autoimmune susceptibility (53). These bacteriophage-driven changes to the bacterial community can in turn alter the availability of MAMPs and immunomodulatory metabolites, indirectly modifying the host’s innate and adaptive immune tone. Unlike fungi and bacteria, which directly engage host PRRs, bacteriophages modulate autoimmunity predominantly through indirect mechanisms by restructuring bacterial communities and altering their immunogenic properties.

Specific disease-related evidence further contextualizes these inter-kingdom dynamics. In psoriatic lesions, *Malassezia* spp. elicit cutaneous inflammation through lipolytic metabolism, concurrently activating the IL-23/IL-17 signaling axis to accelerate disease pathogenesis (54). SLE patients exhibit elevated intestinal phage abundance, which potentially induces dysregulated immune cell activation through IFN-α pathway stimulation (55). In MS, endogenous retroviral elements such as HERV-W can activate pattern recognition receptors, triggering neuronal apoptosis. Meanwhile, children genetically predisposed to T1D show heightened *Enteroviridae* levels in the gut, a phenomenon linked to cross-reactive T cell attacks on pancreatic β-cells through molecular mimicry mechanisms (56). Collectively, these discoveries delineate the bidirectional modulation within intestinal mycobiome-virome-bacteriome interaction networks during AD pathogenesis, providing a conceptual framework for mechanistic exploration and development of targeted therapeutic modalities.

While disease-specific perturbations in the gut mycobiome and virome have been linked to ADs and other immune-mediated disorders, the identification and validation of non-bacterial microbiome biomarkers face unique methodological hurdles that must be overcome to advance translational research. Notably, low biomass of fungi and viruses in clinical samples often leads to insufficient sequencing coverage (53); potential contamination during sample collection and processing can skew results (57); incomplete reference databases for non-bacterial taxa limit accurate identification (58); and inherent sequencing biases may misrepresent the true composition of the mycobiome and virome (59). Accordingly, non-bacterial microbiome biomarkers should be interpreted with caution, and their validity must be verified using orthogonal approaches across independent cohorts to ensure reliability. Addressing these gaps through standardized multi-kingdom sequencing protocols, expanded fungal and viral reference genomes, and integrated bioinformatics pipelines will be essential for unlocking the full translational potential of non-bacterial biomarkers. These limitations not only hinder the discovery of robust non-bacterial biomarkers but also contribute to the overrepresentation of bacteria-focused studies in the field, perpetuating the imbalance in our understanding of microbiome contributions to ADs.

Together, these inter-kingdom dynamics demonstrate that the microbiome functions as an integrated multi-domain ecosystem rather than a collection of isolated kingdoms. Fungi and viruses act as critical amplifiers or modulators of autoimmune inflammation, reinforcing the need for a holistic, systems-level view of the microbiome in future biomarker discovery and therapeutic development.

## 3 Definition and classification of microbiome biomarkers

In this review, we define microbiome biomarkers as measurable microbial or microbiome-associated features that reflect disease susceptibility, activity, prognosis, or therapeutic response in ADs. Based on their biological origin, these biomarkers can be categorized into three mutually inclusive classes: taxonomic, functional/metabolic, and host-microbiome interaction-derived biomarkers, each exhibiting distinct mechanistic relevance to ADs.

### 3.1 Core definition and classification of microbiome biomarkers

As introduced earlier, we define microbiome biomarkers as measurable microbial or microbiome-associated features that reflect disease susceptibility, activity, prognosis, or therapeutic response in ADs. These biomarkers can be categorized into three classes, each with unique mechanistic relevance to ADs, as systematically summarized in **Table 3**.

**Table 3:** Classification and representative examples of microbiome biomarkers in ADs.

Category	Definition	Representative examples	Mechanistic links to autoimmunity	References
Taxonomic biomarkers	Disease-associated shifts in microbial composition at the phylum, genus, or species level.	1. *Prevotella copri* (RA); 2. *Ruminococcus gnavus* (SLE); 3. *Bifidobacterium* spp. (T1D).	1. *Prevotella copri* is highly enriched in newly-onset RA with elevated disease activity, and its abundance decreases as clinical remission is achieved; 2. *Ruminococcus gnavus* drives anti-dsDNA production via molecular mimicry; 3. reduced *Bifidobacterium* precedes T1D onset.	(11, 20, 21, 23)
Functional/metabolic biomarkers	Microbial genes, pathways, enzymes, and metabolites mediating host–microbiome crosstalk.	1. SCFAs; 2. bile acid derivatives; 3. tryptophan catabolites.	1. SCFAs enhance Treg differentiation and epithelial barrier integrity; 2. bile acids attenuate inflammation via NF-κB inhibition; 3. tryptophan catabolites modulate innate immune sensing.	(12, 62)
Host–microbiome interaction-derived biomarkers	Host immune signatures induced by microbial components or dysbiosis	1. PRR activation (TLRs/NLRs/CLRs); 2. cytokine profile shifts 3. anti-commensal antibodies.	1. MAMPs activate PRRs to trigger inflammatory cascades; 2. dysbiosis-induced barrier breach releases DAMPs and drives systemic inflammation.	(13–15)

- Taxonomic biomarkers. These refer to disease-associated shifts in microbial composition at the phylum, genus, or species level (11). For example, enrichment of pathobionts or depletion of protective commensals directly reflects the dysbiotic state and correlates with disease activity (60, 61).
- Functional/metabolic biomarkers. These include microbial genes, signaling pathways, enzymes, and metabolites that mediate host-microbe crosstalk (12). Typical representatives include SCFA, bile acid derivatives, and tryptophan catabolites. Such biomarkers can participate in the pathogenesis of ADs by regulating immune homeostasis, epithelial barrier integrity, and inflammatory status (62).
- Host-microbiome interaction-derived biomarkers. These are host immune signatures induced by microbial components or dysbiosis (13). They include the activation of PRRs, changes in cytokine profiles, and adaptive immune responses that can reflect the impact of microbial signals on host functions.

Beyond their roles in ADs, microbiome dynamics have been shown to influence the pathogenesis of a broader range of conditions, including metabolic disorders and malignancies. Taxonomic and functional alterations in microbial communities are now widely recognized as promising biomarker candidates across these contexts. Furthermore, microbiome-derived biomarkers show translational potential in predicting therapeutic efficacy, with specific shifts in microbiota composition demonstrating predictive value for immune checkpoint inhibitors (ICIs) responsiveness, offering novel insights into precision medicine strategies (63).

### 3.2 Mechanistic links between microbiome biomarkers and autoimmunity

From an immunological perspective, microbiome biomarkers are not merely descriptive indicators of dysbiosis. Instead, they often represent the molecular triggers and footprints of innate immune activation that bridge dysbiosis and autoimmunity. Specifically, many microbial taxonomic and metabolic biomarkers can be conceptualized as MAMPs (e.g., lipopolysaccharide, peptidoglycan, β-glucans) that are directly sensed by host PRRs (TLRs, NLRs, CLRs). This recognition activates downstream inflammatory cascades, thereby modulating disease activity. Furthermore, the breach of epithelial barrier integrity during dysbiosis leads to the release of DAMPs from stressed or dying host cells, which further amplify innate immune responses and serve as indirect biomarkers of microbiome-induced tissue injury (14, 15). These processes drive three key mechanisms linking dysbiosis to autoimmunity, and microbiome biomarkers are direct reflections of each.

- Molecular mimicry. Structural homology exists between microbial antigens (e.g., *surface proteins of Klebsiella pneumoniae*) and host self-antigens, which can trigger cross-reactive T-cell responses and autoimmune tissue damage (64).
- Bystander activation. Chronic inflammation induced by dysbiosis can lower the immune activation threshold, resulting in non-specific activation of autoreactive lymphocytes in genetically susceptible individuals (65).
- Systemic microbial translocation. Impaired intestinal epithelial barrier function allows microbial products to enter the systemic circulation, triggering low-grade inflammation and activating innate immune pathways, which further exacerbate autoimmune responses (66).

As research advances on the linkage between the microbiome and ADs, the application potential of microbiome biomarkers in disease surveillance, therapeutic response prediction, and early disease warning is gradually being recognized. In terms of disease surveillance, the structure of the gut microbiota is dynamically correlated with the activity of ADs, which can serve as an important indicator for evaluating disease progression.

## 4 Application of microbiome biomarkers in ADs

### 4.1 The potential for early diagnosis

Microbiome biomarkers have demonstrated significant potential for early detection of ADs. Emerging evidence indicates that gut microbiome composition is closely linked to the pathogenesis of various ADs. Notably, structural alterations in specific microbial communities may serve as predictive biomarkers for early disease risk. Distinct microbial signatures have been identified as potential diagnostic biomarkers for SLE and RA. Gut microbiome profiling in RA patients consistently shows a surge in pathobionts like *Prevotella copri* and a decline in beneficial commensals like *Faecalibacterium prausnitzii*. This disturbance directly correlates with disease severity (20, 67, 68). SLE patients exhibit reduced abundance of *Lactobacillus* and *Bifidobacterium*, alongside an elevated presence of *Ruminococcus gnavus*, which correlates with anti-dsDNA antibody titers (21, 69, 70). Longitudinal microbiome monitoring offers novel approaches for early disease detection. Longitudinal monitoring of microbiome dynamics allows early identification of disease risk markers, thereby supporting timely preventive intervention in clinical practice.

Through systematic analysis of patient microbiome samples, researchers have identified characteristic microbial communities associated with disease phenotypes, thereby facilitating the development of microbiome-based diagnostics. These diagnostic platforms enable precise detection of early-stage pathologies while offering clinicians molecular evidence to establish patient-specific treatment regimens, ultimately enhancing therapeutic efficacy and clinical prognosis.

### 4.2 Disease monitoring and prognosis assessment

Microbiome biomarkers demonstrate dual clinical utility in monitoring ADs and evaluating disease prognosis. Multivariate analyses reveal significant positive correlations between microbiome structural integrity and both disease activity scores and histopathological severity markers. Longitudinal microbiota profiling demonstrates that compositional dynamics serve as quantitative biomarkers for treatment efficacy assessment. Cohort data analysis identifies temporal synchrony between microbiome homeostasis restoration and clinically validated symptom remission indices. In the biologics-treated IBD cohort, microbiome diversity exhibited a progressive increase that paralleled clinical remission trajectories (71). These findings establish microbiome profiling as a dual-functional tool for real-time disease monitoring and therapeutic response assessment, supporting data-driven personalized treatment strategies.

### 4.3 Evidence gaps and barriers to clinical implementation

#### 4.3.1 Evidence grading of microbiome biomarkers

A realistic appraisal reveals considerable heterogeneity in the clinical readiness of candidate biomarkers. A subset of proposed markers has been consistently reproduced across multiple independent cohorts with rigorous confounder control. For example, the enrichment of *Prevotella copri* in RA and the depletion of *Faecalibacterium prausnitzii* in IBD are consistently reproducible across studies and thus carry the strongest translational potential (20, 46). However, most metabolite-based signatures and emerging mycobiome and virome candidates derive from well-designed but single-cohort or modestly sized studies and await independent replication before clinical application (50, 57). The majority of non-bacterial microbiome biomarkers remain at an even earlier stage, having been identified in small cross-sectional studies without adequate confounding control or external validation (52, 58). This evidence landscape underscores the need for realistic expectations: only a minority of currently proposed microbiome biomarkers are ready for prospective clinical testing, while most require substantial additional validation.

#### 4.3.2 Shared versus disease-specific signatures

Alongside this variation in evidence strength, comparative analysis reveals both common and distinct microbial signatures across ADs. Reduced SCFA-producing bacteria, especially *Faecalibacterium prausnitzii*, are a recurrent feature in IBD, RA, and MS (18, 72), alongside impaired intestinal barrier integrity (18, 39, 40, 72). Conversely, *Prevotella copri* enrichment is most consistently reported in RA, while *Ruminococcus gnavus* blooms are strongly associated with SLE (20, 21). Whether these apparently disease-specific signatures reflect true pathophysiological differences or the uneven depth of investigation across ADs remains an open question, as IBD, RA, and SLE are far more extensively profiled than autoimmune liver disease or Graves’ disease.

#### 4.3.3 Barriers to clinical implementation

Beyond the evidence gaps noted above, methodological standardization remains a critical bottleneck. Divergent choices in 16S rRNA variable regions, sequencing depths, and bioinformatic pipelines generate inconsistent results across studies, impeding the establishment of clinical reference ranges. From a regulatory perspective, frameworks for microbiome-based diagnostics are nascent: certified reference materials, quality control metrics, and consensus definitions of normal versus dysbiotic states are lacking. Addressing these barriers will require multi-site analytical validation, development of microbiome standards, engagement with regulatory agencies, and prospective trials linking biomarker-guided interventions to clinical outcomes.

### 4.4 Individualized treatment strategy

#### 4.4.1 Probiotics/prebiotics

Probiotics and prebiotics, as pivotal therapeutic strategies for microbiome-targeted interventions, have demonstrated significant clinical efficacy in the management of various ADs (73). Probiotics modulate gut microbiota through direct supplementation of viable beneficial microorganisms, whereas prebiotics stimulate selective growth of commensal bacteria via specialized nutritional substrates. Synbiotics, synergistic combinations of probiotics and prebiotics, enhance therapeutic outcomes through dual mechanisms, replenishing beneficial microbial populations and providing targeted metabolic substrates. These interventions exhibit therapeutic potential by remodeling microbial ecology, reinforcing intestinal epithelial integrity, and restoring immune homeostasis (74). Clinical evidence indicates that the multi-strain probiotic formulation VSL#3 ameliorates IBD by downregulating key pro-inflammatory mediators, such as IL-6 and TNF-α, while simultaneously upregulating intestinal barrier proteins (75). It should be noted that the post-2016 VSL#3 probiotic formulation differs from the De Simone Formulation, which was commercially available under the trademark VSL#3 only until 2016 (76). Furthermore, Lactobacillus plantarum 299v demonstrates the ability to improve survival outcomes and attenuate renal immunopathology in SLE patients via the generation of AhR ligands through tryptophan metabolism and modulation of plasma cell differentiation (77). Specific prebiotics (e.g., oligofructose, resistant starch, and fucooligosaccharides) exert synergistic effects by promoting beneficial bacterial proliferation, elevating SCFA levels, and improving intestinal barrier function (78). Preclinical studies show that engineered probiotics can rebalance pro- and anti-inflammatory cytokine profiles, alleviate oxidative stress, and restore gut microbial composition in murine IBD models. However, clinical translation remains constrained by transient gut colonization capacity, horizontal gene transfer risks, and host-specific variability, necessitating cautious therapeutic application (79).

While probiotics and prebiotics demonstrate therapeutic promise, they encounter three primary challenges: strain specificity, individual heterogeneity, and delivery system limitations. To address these constraints, future investigations should prioritize interconnected strategies: precision screening of microbial strains, development of personalized drug delivery regimens, and optimization of targeted delivery mechanisms to enhance gut colonization efficiency. Such advances can improve the efficacy of microbial therapeutics, accelerate their clinical translation for the management of ADs, and ultimately promote precision medicine via novel microbiome-based therapeutic strategies.

#### 4.4.2 Fecal microbiota transplantation

Fecal microbiota transplantation (FMT), an innovative therapeutic approach, restores gut microbiota homeostasis via the transfer of a complex microbial community from a healthy donor, offering novel treatment strategies for refractory ADs. The therapeutic mechanisms of FMT involve enhancing microbial diversity, enriching beneficial taxa such as *Faecalibacterium prausnitzii* and *Roseburia* spp., suppressing the expansion of pathogenic *Enterobacteriaceae*, and restoring balanced host-microbiota crosstalk. Functionally, FMT promotes Treg differentiation through SCFA-mediated activation of the GPR43 signaling pathway while concurrently suppressing excessive Th17 and Th1 activation to restore immune homeostasis (80). Furthermore, secondary bile acids produced by the engrafted microbiota attenuate tissue inflammation via inhibition of the NF-κB signaling pathway, while inducing favorable metabolic reprogramming in host immune cells (81). Mounting clinical evidence supports the therapeutic potential of FMT across multiple autoimmune conditions, including IBD, RA, MS, and SLE (82). Notably, several randomized controlled trials have shown efficacy for FMT in the treatment of ulcerative colitis (83).

However, FMT faces substantial challenges, including inconsistent donor selection criteria, suboptimal delivery strategies, and unresolved long-term safety concerns (84). Critically, robust long-term safety data remain limited, as most studies only report short-term follow-up (85). The potential for delayed adverse events has not been systematically evaluated, including the theoretical risk of transmitting undiagnosed metabolic predispositions, autoimmune susceptibility, or other unintended donor phenotypes (86). Optimal donor profiles require high microbial diversity, high SCFA-producing capacity, and the absence of autoimmune disease predisposition. Compared with allogeneic FMT, autologous FMT may offer advantages in terms of immunological compatibility (87). Additionally, the lack of standardized manufacturing protocols and clear regulatory frameworks continues to hinder the reproducibility and scalability of FMT-based therapies. Technological advances in encapsulation and mucosa-targeted delivery systems improve microbial engraftment efficiency by protecting microbes from gastrointestinal degradation (88). Although short-term outcomes are primarily characterized by transient, mild adverse events, longitudinal surveillance protocols are needed to address potential pathogen transmission risks through multi-tiered screening, including shotgun metagenomic sequencing, metabolomic profiling, and human leukocyte antigen (HLA) compatibility assessment.

While FMT demonstrates promising therapeutic potential for refractory ADs, further refinement through integrated multi-omics monitoring platforms and optimized delivery systems is essential to establish evidence-based clinical guidelines. Therefore, FMT should currently be considered an exploratory intervention rather than a broadly established treatment for ADs. The field is increasingly shifting toward defined microbial consortia, next-generation probiotics, and targeted metabolite-based interventions, which may offer improved reproducibility, safety, and regulatory feasibility compared with conventional FMT.

## 5 Discussion

Elucidating the intricate interactions between the microbiome and the host immune system constitutes a cornerstone of contemporary biomedical research, offering a paradigm shift in our understanding of the pathogenesis of ADs. This review has synthesized compelling evidence that individual variations in microbiome composition and function are key determinants of immune heterogeneity, influencing disease susceptibility, progression, and therapeutic responses. The identification of specific microbial signatures and their mechanisms of action, as detailed in the previous sections, not only deepens our mechanistic understanding but also challenges conventional disease taxonomies, establishing an empirical basis for precision medicine frameworks. Integrative analysis leveraging multi-omics technologies is now enabling the translation of these discoveries into clinically actionable biomarker panels and novel therapeutic modalities.

Substantial advances notwithstanding, persistent scientific challenges impede translational progress. The inherent complexity of microbial ecosystems combined with interindividual variability compromises the reproducibility and external validity of research findings. In addition, methodological inconsistencies in experimental design and analytical workflows critically hinder the clinical implementation of microbiome-based biomarkers. It remains a subject of active debate whether dysbiosis is a primary driver of autoimmunity, a secondary consequence of inflammation, or a perpetuating factor. Furthermore, the context-dependent role of certain microbes presents a significant challenge.

Despite accumulating evidence, the high inter-individual variability in microbiome signatures mandates a critical reappraisal of confounders that shape microbial communities independently of disease. Three factors merit particular attention.

- Diet. Dietary patterns are among the strongest determinants of gut microbiome composition. Long-term macronutrient intake, fiber content, and food additives influence both taxonomic and metabolic outputs, often overlapping with disease-associated signatures. For example, a high-fat, low-fiber diet can reduce SCFA production and promote intestinal permeability, mimicking the functional dysbiosis observed in IBD and RA. Of the studies cited in this review, only a subset controlled for dietary intake through food frequency questionnaires or standardized dietary periods (8, 18, 20, 45, 89), whereas most cross-sectional studies did not adequately account for diet. This raises the possibility that some reported “disease signatures” may partially reflect dietary differences between patients and controls rather than disease-specific alterations.
- Geographic origin and ethnicity. The human microbiome exhibits pronounced biogeographic variation, with distinct enterotypes and microbial gene catalogues associated with different continents and ethnic groups. A *Prevotella*-dominated enterotype, for instance, is more prevalent in populations consuming plant-based diets in sub-Saharan Africa and parts of Asia, which overlaps with the *Prevotella copri* enrichment reported in RA. Failure to match cases and controls by geography and ethnicity can introduce significant bias, particularly in meta-analyses that pool diverse cohorts. Among the studies reviewed, several performed stratified analyses or included geographic origin as a covariate (8, 20, 23, 45, 46), yet many single-center investigations with limited demographic scope did not explicitly address this confounder.
- Medication and therapeutic interventions. Medications commonly used in ADs, including glucocorticoids, methotrexate, biological DMARDs, and proton pump inhibitors, exert substantial and often rapid effects on the gut microbiota. For example, methotrexate has been shown to reduce the abundance of Bacteroidetes, an effect that parallels and potentially confounds the microbial signatures attributed to RA itself. Additionally, antibiotic exposure preceding sample collection can cause sustained disruptions that mask or mimic disease-related dysbiosis. A critical review of the literature reveals that while some recent studies adjusted for medication use in multivariate models or employed treatment-naïve cohorts (22, 35, 45, 46, 63), many earlier investigations did not comprehensively document or control for pharmacological exposures. Consequently, discerning treatment-induced microbial remodeling from primary disease-associated dysbiosis remains a major challenge. These confounders converge to limit the reproducibility and generalizability of microbiome biomarker studies.

The clinical translation of microbiome research faces significant hurdles rooted in methodological and biological complexity. The lack of standardized protocols for sample collection, DNA extraction, sequencing, and bioinformatic analysis compromises the reproducibility and cross-study comparability of findings. Moreover, the high degree of inter-individual variability in microbiome composition, influenced by genetics, diet, geography, and medication, poses a major obstacle to defining universal diagnostic benchmarks or therapeutic interventions. Current microbiome-based interventions, such as probiotics and FMT, show promise but are often characterized by inconsistent efficacy, likely due to strain-specific effects, the resilience of the indigenous gut microbiota, and the failure to account for individual patient context. Addressing these barriers will require multi-center harmonization studies, development of microbiome reference standards, and engagement with regulatory bodies.

## 6 Future perspectives and standardization needs

Looking forward, bridging these gaps requires a concerted multi-disciplinary effort. Several key priorities emerge from this review.

- Longitudinal and causal studies. Large-scale, longitudinal cohort studies from diverse populations are needed to track microbiome dynamics before and after disease onset, helping to distinguish cause from effect. Advanced techniques like gnotobiotic models, microbial metabolome tracing, and humanized mice will be crucial for establishing mechanistic causality. Only through such designs can the field move beyond correlational observations toward actionable causal insights.
- Standardization and benchmarking. The development of standardized, end-to-end pipelines for microbiome analysis is a non-negotiable prerequisite for the field to progress. This includes creating validated reference databases and standardized bioinformatics frameworks. International collaborative initiatives are needed to develop certified reference materials and proficiency testing programs, without which microbiome-based diagnostics cannot achieve the analytical rigor expected of clinical-grade assays.
- From correlation to mechanism. Moving beyond taxonomic census to functional understanding is critical. This requires a deeper focus on meta-transcriptomics, proteomics, and metabolomics to characterize the active functional pathways and the key microbial metabolites that mediate immune modulation. Bioinformatics and artificial intelligence (AI) have become integral to advancing microbiome research beyond descriptive taxonomic profiling, driving the transition toward predictive, mechanistic, and translational applications. First, machine learning algorithms address the inherent complexity of high-dimensional microbiome datasets by enabling robust feature selection, identifying reproducible microbial biomarkers associated with disease risk, activity, and treatment response. These methods, including random forests, support vector machines, and LASSO regression, help filter noise and prioritize biologically meaningful taxa or functional pathways that may serve as early diagnostic or prognostic indicators. Second, multi-omics integration frameworks, such as multi-omics factor analysis (MOFA) and deep learning architectures, enable the integration of taxonomic, metagenomic, transcriptomic, metabolomic, and host immune readouts into cohesive, mechanistic networks. These tools reveal how microbial communities interact with host physiology, uncovering key regulatory nodes that drive autoimmune pathogenesis, such as microbial metabolite-host receptor crosstalk and immune-metabolic feedback loops. Third, explainable AI (XAI) methods applied to predictive models are critical for clinical validation, as they reveal which specific microbial features (e.g., butyrate-producing taxa, lipopolysaccharide biosynthetic pathways) directly influence disease risk or treatment outcomes, rather than providing black-box predictions. Rigorous cross-validation across independent cohorts, external testing in diverse populations, and explicit attention to model interpretability and generalizability remain essential to ensure these computational tools translate reliably into clinical practice, avoiding overfitting and addressing the inherent heterogeneity of the gut microbiome. Together, these advances move the field beyond purely correlative, taxonomic descriptions toward data-driven, actionable insights that support the development of precision microbiome-based therapies and diagnostic strategies for ADs.
- Precision microbiome engineering. The future of microbiome-based therapeutics lies in precision interventions. This includes the development of defined consortia of bacteria (synthetic microbial communities) rather than generic probiotics, the use of engineered bacteria to deliver therapeutic payloads, and the targeted modulation of specific microbial pathways. These approaches offer greater reproducibility, safety, and regulatory feasibility than conventional FMT.
- Clinical trials and regulatory pathways. Prospective, multi-center clinical trials with harmonized protocols are urgently needed to validate candidate microbiome biomarkers and microbiota-directed interventions. Concurrently, engagement with regulatory agencies is essential to define evidentiary standards for microbiome-based diagnostics and therapeutics, establish frameworks for quality control, and develop guidelines for donor screening and long-term safety monitoring in FMT and related interventions.

In conclusion, while the path forward is complex, the convergence of multi-omics technologies, artificial intelligence, and systems biology holds immense promise. AI algorithms can integrate complex, multi-dimensional data to identify robust biomarkers, predict disease risk, and personalize treatment strategies. By developing sophisticated cross-omics integration platforms and dynamic predictive models, we anticipate the construction of a high-resolution microbiome-immunity interaction atlas. This atlas will not only decode the etiopathogenesis of ADs but will also fundamentally transform diagnostic-therapeutic paradigms, ultimately facilitating the transition from population-based medicine to truly individualized, microbiota-informed healthcare.

## References

[1] Yu R Zhang C Yuan M Ye S Hu T Chen S . Exercise-induced metabolite N-lactoyl-phenylalanine ameliorates colitis by inhibiting M1 macrophage polarization via the suppression of the Nf-kb signaling pathway. Cell Mol Gastroenterol Hepatol. (2025) 19:101558. doi: 10.1016/j.jcmgh.2025.101558 40562095 PMC12391280

[2] Yuan M Chen S Lin Z Yu R Chao K Ye S . Acss2-mediated histone H4 lysine 12 crotonylation (H4k12cr) alleviates colitis via enhancing transcription of Cldn7. Adv Sci (Weinh). (2025) 12:e00461. doi: 10.1002/advs.202500461 40650658 PMC12376547

[3] Yuan M Li S Chen S Zhu M Yu R Huang J . Acss2-mediated lysine crotonylation attenuates senescence and enhances the therapeutic efficacy of adipose-derived stem cells in inflammatory bowel disease. Cell Regener. (2026) 15:11. doi: 10.1186/s13619-026-00285-x 41764109 PMC12950121

[4] Tian Y Zhang J Wu L Zhang C Zheng F Yang Y . Tissue-resident memory T cells in rheumatoid immune diseases: Pathogenic mechanisms and therapeutic strategies. Biomedicines. (2025) 13:2945. doi: 10.3390/biomedicines13122945 41462958 PMC12730211

[5] Abend AH He I Bahroos N Christianakis S Crew AB Wise LM . Estimation of prevalence of autoimmune diseases in the United States using electronic health record data. J Clin Invest. (2024) 135:e178722. doi: 10.1172/jci178722 39666393 PMC11827834

[6] Conrad N Misra S Verbakel JY Verbeke G Molenberghs G Taylor PN . Incidence, prevalence, and co-occurrence of autoimmune disorders over time and by age, sex, and socioeconomic status: A population-based cohort study of 22 million individuals in the UK. Lancet. (2023) 401:1878–90. doi: 10.1016/s0140-6736(23)00457-9 37156255

[7] Schattner A . Unusual presentations of systemic lupus erythematosus: A narrative review. Am J Med. (2022) 135:1178–87. doi: 10.1016/j.amjmed.2022.05.020 35671786

[8] Cho I Blaser MJ . The human microbiome: At the interface of health and disease. Nat Rev Genet. (2012) 13:260–70. doi: 10.1038/nrg3182 22411464 PMC3418802

[9] De Luca F Shoenfeld Y . The microbiome in autoimmune diseases. Clin Exp Immunol. (2019) 195:74–85. doi: 10.1111/cei.13158 29920643 PMC6300652

[10] Felix KM Tahsin S Wu HJ . Host-microbiota interplay in mediating immune disorders. Ann N Y Acad Sci. (2018) 1417:57–70. doi: 10.1111/nyas.13508 28984367 PMC5889363

[11] Zhou H Balint D Shi Q Vartanian T Kriegel MA Brito I . Lupus and inflammatory bowel disease share a common set of microbiome features distinct from other autoimmune disorders. Ann Rheum Dis. (2025) 84:93–105. doi: 10.1136/ard-2024-225829 39874239 PMC11868722

[12] Shtossel O Koren O Shai I Rinott E Louzoun Y . Gut microbiome-metabolome interactions predict host condition. Microbiome. (2024) 12:24. doi: 10.1186/s40168-023-01737-1 38336867 PMC10858481

[13] Berryman MA Ilonen J Triplett EW Ludvigsson J . Important denominator between autoimmune comorbidities: A review of class II HLA, autoimmune disease, and the gut. Front Immunol. (2023) 14:1270488. doi: 10.3389/fimmu.2023.1270488 37828987 PMC10566625

[14] Wang X Yuan W Yang C Wang Z Zhang J Xu D . Emerging role of gut microbiota in autoimmune diseases. Front Immunol. (2024) 15:1365554. doi: 10.3389/fimmu.2024.1365554 38765017 PMC11099291

[15] Singh K Bhadauriya AS . Effect of microbial dysbiosis on autoimmune associated inflammation. Int Rev Cell Mol Biol. (2025) 395:1–22. doi: 10.1016/bs.ircmb.2024.12.016 40653352

[16] Suliman BA . Potential clinical implications of molecular mimicry-induced autoimmunity. Immun Inflammation Dis. (2024) 12:e1178. doi: 10.1002/iid3.1178 38415936 PMC10832321

[17] Cheok YY Tan GMY Lee CYQ Abdullah S Looi CY Wong WF . Innate immunity crosstalk with Helicobacter pylori: Pattern recognition receptors and cellular responses. Int J Mol Sci. (2022) 23:7561. doi: 10.3390/ijms23147561 35886908 PMC9317022

[18] Mann ER Lam YK Uhlig HH . Short-chain fatty acids: Linking diet, the microbiome and immunity. Nat Rev Immunol. (2024) 24:577–95. doi: 10.1038/s41577-024-01014-8 38565643

[19] Yoon JH Do JS Velankanni P Lee CG Kwon HK . Gut microbial metabolites on host immune responses in health and disease. Immune Netw. (2023) 23:e6. doi: 10.4110/in.2023.23.e6 36911800 PMC9995988

[20] Scher JU Sczesnak A Longman RS Segata N Ubeda C Bielski C . Expansion of intestinal Prevotella copri correlates with enhanced susceptibility to arthritis. Elife. (2013) 2:e01202. doi: 10.7554/eLife.01202 24192039 PMC3816614

[21] Azzouz D Omarbekova A Heguy A Schwudke D Gisch N Rovin BH . Lupus nephritis is linked to disease-activity associated expansions and immunity to a gut commensal. Ann Rheum Dis. (2019) 78:947–56. doi: 10.1136/annrheumdis-2018-214856 30782585 PMC6585303

[22] Artacho A Isaac S Nayak R Flor-Duro A Alexander M Koo I . The pretreatment gut microbiome is associated with lack of response to methotrexate in new-onset rheumatoid arthritis. Arthritis Rheumatol. (2021) 73:931–42. doi: 10.1002/art.41622 33314800 PMC11293279

[23] Vatanen T Franzosa EA Schwager R Tripathi S Arthur TD Vehik K . The human gut microbiome in early-onset type 1 diabetes from the TEDDY study. Nature. (2018) 562:589–94. doi: 10.1038/s41586-018-0620-2 30356183 PMC6296767

[24] Kriegel MA Sefik E Hill JA Wu HJ Benoist C Mathis D . Naturally transmitted segmented filamentous bacteria segregate with diabetes protection in nonobese diabetic mice. Proc Natl Acad Sci USA. (2011) 108:11548–53. doi: 10.1073/pnas.1108924108 21709219 PMC3136249

[25] Chen R Zou J Chen J Zhong X Kang R Tang D . Pattern recognition receptors: Function, regulation and therapeutic potential. Signal Transd Targ Ther. (2025) 10:216. doi: 10.1038/s41392-025-02264-1 40640149 PMC12246121

[26] Schären OP Hapfelmeier S . Robust microbe immune recognition in the intestinal mucosa. Genes Immun. (2021) 22:268–75. doi: 10.1038/s41435-021-00131-x 33958733 PMC8497264

[27] Furnari S Ciantia R Garozzo A Furneri PM Fuochi V . Lactobacilli-derived microbe-associated molecular patterns (Mamps) in host immune modulation. Biomolecules. (2025) 15:1609. doi: 10.3390/biom15111609 41301527 PMC12650587

[28] Shao T Hsu R Rafizadeh DL Wang L Bowlus CL Kumar N . The gut ecosystem and immune tolerance. J Autoimmun. (2023) 141:103114. doi: 10.1016/j.jaut.2023.103114 37748979

[29] Azzouz DF Chen Z Izmirly PM Chen LA Li Z Zhang C . Longitudinal gut microbiome analyses and blooms of pathogenic strains during lupus disease flares. Ann Rheum Dis. (2023) 82:1315–27. doi: 10.1136/ard-2023-223929 37365013 PMC10511964

[30] Chen S Luo Y Wei G Liu S . Molecular mimicry at the gut-immune interface: A mechanistic link to type 1 diabetes. Immunology. (2026) 177:701–12. doi: 10.1111/imm.70091 41461600

[31] Yi YS Kim HG Kim JH Yang WS Kim E Jeong D . Syk-Myd88 axis is a critical determinant of inflammatory-response in activated macrophages. Front Immunol. (2021) 12:767366. doi: 10.3389/fimmu.2021.767366 35003083 PMC8733199

[32] Li M Zhang R Li J Li J . The role of C-type lectin receptor signaling in the intestinal microbiota-inflammation-cancer axis. Front Immunol. (2022) 13:894445. doi: 10.3389/fimmu.2022.894445 35619716 PMC9127077

[33] Kang N Ji Z Li Y Gao J Wu X Zhang X . Metabolite-derived damage-associated molecular patterns in immunological diseases. FEBS J. (2024) 291:2051–67. doi: 10.1111/febs.16902 37432883

[34] Liu Q He W Tang R Ma X . Intestinal homeostasis in autoimmune liver diseases. Chin Med J (Engl). (2022) 135:1642–52. doi: 10.1097/cm9.0000000000002291 36193976 PMC9509077

[35] Qi XY Liu MX Jiang XJ Gao T Xu GQ Zhang HY . Gut microbiota in rheumatoid arthritis: Mechanistic insights, clinical biomarkers, and translational perspectives. Autoimmun Rev. (2025) 24:103912. doi: 10.1016/j.autrev.2025.103912 40825448

[36] Zhao H Lin G Yin Y Wu Q Wang Y Tang N . Impact of micro- and nanoplastics on gastrointestinal diseases: Recent advances. Eur J Intern Med. (2025) 139:106419. doi: 10.1016/j.ejim.2025.07.015 40701881

[37] Ullah H Arbab S Tian Y Chen Y Liu CQ Li Q . Crosstalk between gut microbiota and host immune system and its response to traumatic injury. Front Immunol. (2024) 15:1413485. doi: 10.3389/fimmu.2024.1413485 39144142 PMC11321976

[38] Di Sabatino A Santacroce G Rossi CM Broglio G Lenti MV . Role of mucosal immunity and epithelial-vascular barrier in modulating gut homeostasis. Intern Emerg Med. (2023) 18:1635–46. doi: 10.1007/s11739-023-03329-1 37402104 PMC10504119

[39] An J Liu Y Wang Y Fan R Hu X Zhang F . The role of intestinal mucosal barrier in autoimmune disease: A potential target. Front Immunol. (2022) 13:871713. doi: 10.3389/fimmu.2022.871713 35844539 PMC9284064

[40] Fasano A . Zonulin and its regulation of intestinal barrier function: The biological door to inflammation, autoimmunity, and cancer. Physiol Rev. (2011) 91:151–75. doi: 10.1152/physrev.00003.2008 21248165

[41] Garcia AC Six N Ma L Morel L . Intersection of the microbiome and immune metabolism in lupus. Immunol Rev. (2024) 325:77–89. doi: 10.1111/imr.13360 38873851 PMC11338729

[42] Qi P Chen X Tian J Zhong K Qi Z Li M . The gut homeostasis-immune system axis: Novel insights into rheumatoid arthritis pathogenesis and treatment. Front Immunol. (2024) 15:1482214. doi: 10.3389/fimmu.2024.1482214 39391302 PMC11464316

[43] Xin X Wang Q Qing J Song W Gui Y Li X . Th17 cells in primary Sjögren's syndrome negatively correlate with increased Roseburia and Coprococcus. Front Immunol. (2022) 13:974648. doi: 10.3389/fimmu.2022.974648 36275752 PMC9579428

[44] Lei Y Liu Q Li Q Zhao C Zhao M Lu Q . Exploring the complex relationship between microbiota and systemic lupus erythematosus. Curr Rheumatol Rep. (2023) 25:107–16. doi: 10.1007/s11926-023-01102-z 37083877

[45] Wang H Cai Y Wu W Zhang M Dai Y Wang Q . Exploring the role of gut microbiome in autoimmune diseases: A comprehensive review. Autoimmun Rev. (2024) 23:103654. doi: 10.1016/j.autrev.2024.103654 39384149

[46] Wang Y Wei J Zhang W Doherty M Zhang Y Xie H . Gut dysbiosis in rheumatic diseases: A systematic review and meta-analysis of 92 observational studies. EBioMedicine. (2022) 80:104055. doi: 10.1016/j.ebiom.2022.104055 35594658 PMC9120231

[47] Li L Cai F Liu Z Mo W Zhang J Qin J . Cross-kingdom microbial interactions in the gut during inflammatory bowel disease. J Transl Med. (2026) 24:456. doi: 10.1186/s12967-026-07692-3 41749264 PMC13041150

[48] Zhang F Aschenbrenner D Yoo JY Zuo T . The gut mycobiome in health, disease, and clinical applications in association with the gut bacterial microbiome assembly. Lancet Microbe. (2022) 3:e969–e83. doi: 10.1016/s2666-5247(22)00203-8 36182668

[49] Oh S Li K Prince A Wheeler ML Hamade H Nguyen C . Pathogen size alters C-type lectin receptor signaling in dendritic cells to influence Cd4 Th9 cell differentiation. Cell Rep. (2022) 38:110567. doi: 10.1016/j.celrep.2022.110567 35354044 PMC9052946

[50] Zheng Z Huang Q Kang Y Liu Y Luo W . Different molecular sizes and chain conformations of water-soluble yeast β-glucan fractions and their interactions with receptor Dectin-1. Carbohydr Polym. (2021) 273:118568. doi: 10.1016/j.carbpol.2021.118568 34560979

[51] Wang Y Spatz M Da Costa G Michaudel C Lapiere A Danne C . Deletion of both dectin-1 and dectin-2 affects the bacterial but not fungal gut microbiota and susceptibility to colitis in mice. Microbiome. (2022) 10:91. doi: 10.1186/s40168-022-01273-4 35698210 PMC9195441

[52] Puxty RJ Millard AD . Functional ecology of bacteriophages in the environment. Curr Opin Microbiol. (2023) 71:102245. doi: 10.1016/j.mib.2022.102245 36512900

[53] Liu Y Liao X Chen Q Wang H Dai H . What is the impact of the virome and mycobiome on female reproductive tract health? A systematic scoping review. Front Immunol. (2026) 17:1749584. doi: 10.3389/fimmu.2026.1749584 42039195 PMC13106131

[54] Fang H Hou Y Zhuang H Wang C . The effects of Malassezia in the activation of interleukin (Il)-23/Il-17 axis in psoriasis. New Microbiol. (2022) 45:130–7. 35699562

[55] Chen B Cao J Liu W Zhang Y Liu Y Wang M . Disturbed gut virome with potent interferonogenic property in systemic lupus erythematosus. Sci Bull (Beijing). (2023) 68:295–304. doi: 10.1016/j.scib.2023.01.021 36697300

[56] Krogvold L Edwin B Buanes T Frisk G Skog O Anagandula M . Detection of a low-grade enteroviral infection in the islets of Langerhans of living patients newly diagnosed with type 1 diabetes. Diabetes. (2015) 64:1682–7. doi: 10.2337/db14-1370 25422108

[57] Houshyar Y Zhang F Tavakoli P Grimm MC Hold GL . Neglected kingdoms: The gut virome, mycobiome and their role in inflammatory bowel disease. Gut Microbes. (2026) 18:2653288. doi: 10.1080/19490976.2026.2653288 41928387 PMC13051595

[58] Avershina E Qureshi AI Winther-Larsen HC Rounge TB . Challenges in capturing the mycobiome from shotgun metagenome data: Lack of software and databases. Microbiome. (2025) 13:66. doi: 10.1186/s40168-025-02048-3 40055808 PMC11887097

[59] Boix-Amorós A Monaco H Sambataro E Clemente JC . Novel technologies to characterize and engineer the microbiome in inflammatory bowel disease. Gut Microbes. (2022) 14:2107866. doi: 10.1080/19490976.2022.2107866 36104776 PMC9481095

[60] Federici S Kredo-Russo S Valdés-Mas R Kviatcovsky D Weinstock E Matiuhin Y . Targeted suppression of human Ibd-associated gut microbiota commensals by phage consortia for treatment of intestinal inflammation. Cell. (2022) 185:2879–2898.e24. doi: 10.1016/j.cell.2022.07.003 35931020

[61] Cao X Sun Y Liu H Bi D Zhao L Ding T . Detection of tissue colonizing bacteria and their association with clinicopathological and molecular features of inflammatory bowel disease. J Transl Med. (2025) 23:1107. doi: 10.1186/s12967-025-07095-w 41094525 PMC12522891

[62] Ikeda T Nishida A Yamano M Kimura I . Short-chain fatty acid receptors and gut microbiota as therapeutic targets in metabolic, immune, and neurological diseases. Pharmacol Ther. (2022) 239:108273. doi: 10.1016/j.pharmthera.2022.108273 36057320

[63] Lim MY Hong S Nam YD . Understanding the role of the gut microbiome in solid tumor responses to immune checkpoint inhibitors for personalized therapeutic strategies: A review. Front Immunol. (2024) 15:1512683. doi: 10.3389/fimmu.2024.1512683 39840031 PMC11747443

[64] Dolton G Bulek A Wall A Thomas H Hopkins JR Rius C . Hla a*24:02-restricted T cell receptors cross-recognize bacterial and preproinsulin peptides in type 1 diabetes. J Clin Invest. (2024) 134:e164535. doi: 10.1172/jci164535 39286976 PMC11405051

[65] Rojas M Acosta-Ampudia Y Heuer LS Zang W D MM Ramírez-Santana C . Antigen-specific T cells and autoimmunity. J Autoimmun. (2024) 148:103303. doi: 10.1016/j.jaut.2024.103303 39141985

[66] Martel J Chang SH Ko YF Hwang TL Young JD Ojcius DM . Gut barrier disruption and chronic disease. Trends Endocrinol Metab. (2022) 33:247–65. doi: 10.1016/j.tem.2022.01.002 35151560

[67] Yao Y Cai X Zheng Y Zhang M Fei W Sun D . Short-chain fatty acids regulate B cells differentiation via the Ffa2 receptor to alleviate rheumatoid arthritis. Br J Pharmacol. (2022) 179:4315–29. doi: 10.1111/bph.15852 35393660

[68] Amarnani A Silverman GJ . Understanding the roles of the microbiome in autoimmune rheumatic diseases. Rheumatol Immunol Res. (2023) 4:177–87. doi: 10.2478/rir-2023-0027 38125641 PMC10729600

[69] Liu F Du J Zhai Q Hu J Miller AW Ren T . The bladder microbiome, metabolome, cytokines, and phenotypes in patients with systemic lupus erythematosus. Microbiol Spectr. (2022) 10:e0021222. doi: 10.1128/spectrum.00212-22 35913213 PMC9620774

[70] Shaheen WA Quraishi MN Iqbal TH . Gut microbiome and autoimmune disorders. Clin Exp Immunol. (2022) 209:161–74. doi: 10.1093/cei/uxac057 35652460 PMC9390838

[71] Sorbara MT Pamer EG . Microbiome-based therapeutics. Nat Rev Microbiol. (2022) 20:365–80. doi: 10.1038/s41579-021-00667-9 34992261

[72] Wang Y Roussel-Queval A Chasson L Hanna Kazazian N Marcadet L Nezos A . Tlr7 signaling drives the development of Sjögren's syndrome. Front Immunol. (2021) 12:676010. doi: 10.3389/fimmu.2021.676010 34108972 PMC8183380

[73] Wang R Yu YF Yu WR Sun SY Lei YM Li YX . Roles of probiotics, prebiotics, and postbiotics in B-cell-mediated immune regulation. J Nutr. (2025) 155:37–51. doi: 10.1016/j.tjnut.2024.11.011 39551357

[74] Chen K Wang H Yang X Tang C Hu G Gao Z . Targeting gut microbiota as a therapeutic target in T2dm: A review of multi-target interactions of probiotics, prebiotics, postbiotics, and synbiotics with the intestinal barrier. Pharmacol Res. (2024) 210:107483. doi: 10.1016/j.phrs.2024.107483 39521027

[75] Lorentz A Müller L . Probiotics in the treatment of inflammatory bowel disease in adulthood: A systematic review. J Gastrointestin Liver Dis. (2022) 31:74–84. doi: 10.15403/jgld-3936 35306546

[76] De Simone C . Letter: What gastroenterologists should know about Vsl#3. Aliment Pharmacol Ther. (2018) 47:698–9. doi: 10.1111/apt.14515 29417631

[77] Goh RC Maharajan MK Gopinath D Fang CM . The therapeutic effects of probiotic on systemic lupus erythematosus in lupus mice models: A systematic review. Probiotics Antimicrob Proteins. (2025) 17:35–50. doi: 10.1007/s12602-024-10297-1 38806966

[78] Costa GT Vasconcelos Q Aragão GF . Fructooligosaccharides on inflammation, immunomodulation, oxidative stress, and gut immune response: A systematic review. Nutr Rev. (2022) 80:709–22. doi: 10.1093/nutrit/nuab115 34966938

[79] Zhou J Li M Chen Q Li X Chen L Dong Z . Programmable probiotics modulate inflammation and gut microbiota for inflammatory bowel disease treatment after effective oral delivery. Nat Commun. (2022) 13:3432. doi: 10.1038/s41467-022-31171-0 35701435 PMC9198027

[80] Paramsothy S Kamm MA Kaakoush NO Walsh AJ van den Bogaerde J Samuel D . Multidonor intensive faecal microbiota transplantation for active ulcerative colitis: A randomised placebo-controlled trial. Lancet. (2017) 389:1218–28. doi: 10.1016/s0140-6736(17)30182-4 28214091

[81] Fleishman JS Kumar S . Bile acid metabolism and signaling in health and disease: Molecular mechanisms and therapeutic targets. Signal Transd Targ Ther. (2024) 9:97. doi: 10.1038/s41392-024-01811-6 38664391 PMC11045871

[82] Zeng L Deng Y Yang K Chen J He Q Chen H . Safety and efficacy of fecal microbiota transplantation for autoimmune diseases and autoinflammatory diseases: A systematic review and meta-analysis. Front Immunol. (2022) 13:944387. doi: 10.3389/fimmu.2022.944387 36248877 PMC9562921

[83] Lima SF Gogokhia L Viladomiu M Chou L Putzel G Jin WB . Transferable immunoglobulin a-coated Odoribacter splanchnicus in responders to fecal microbiota transplantation for ulcerative colitis limits colonic inflammation. Gastroenterology. (2022) 162:166–78. doi: 10.1053/j.gastro.2021.09.061 34606847 PMC8678328

[84] He R Li P Wang J Cui B Zhang F Zhao F . The interplay of gut microbiota between donors and recipients determines the efficacy of fecal microbiota transplantation. Gut Microbes. (2022) 14:2100197. doi: 10.1080/19490976.2022.2100197 35854629 PMC9302524

[85] Yau YK Lau LHS Lui RNS Wong SH Guo CL Mak JWY . Long-term safety outcomes of fecal microbiota transplantation: Real-world data over 8 years from the Hong Kong Fmt Registry. Clin Gastroenterol Hepatol. (2024) 22:611–620.e12. doi: 10.1016/j.cgh.2023.09.001 37734581

[86] Cold F Svensson CK Petersen AM Hansen LH Helms M . Long-term safety following faecal microbiota transplantation as a treatment for recurrent Clostridioides difficile infection compared with patients treated with a fixed bacterial mixture: Results from a retrospective cohort study. Cells. (2022) 11:435. doi: 10.3390/cells11030435 35159245 PMC8834574

[87] Santana AB Souto BS Santos NCM Pereira JA Tagliati CA Novaes RD . Murine response to the opportunistic bacterium Pseudomonas aeruginosa infection in gut dysbiosis caused by 5-fluorouracil chemotherapy-induced mucositis. Life Sci. (2022) 307:120890. doi: 10.1016/j.lfs.2022.120890 35988752

[88] Wang Y Zhang S Borody TJ Zhang F . Encyclopedia of fecal microbiota transplantation: A review of effectiveness in the treatment of 85 diseases. Chin Med J (Engl). (2022) 135:1927–39. doi: 10.1097/cm9.0000000000002339 36103991 PMC9746749

[89] Kostic AD Gevers D Siljander H Vatanen T Hyötyläinen T Hämäläinen AM . The dynamics of the human infant gut microbiome in development and in progression toward type 1 diabetes. Cell Host Microbe. (2015) 17:260–73. doi: 10.1016/j.chom.2015.01.001 25662751 PMC4689191

[90] Greiling TM Dehner C Chen X Hughes K Iñiguez AJ Boccitto M . Commensal orthologs of the human autoantigen Ro60 as triggers of autoimmunity in lupus. Sci Transl Med. (2018) 10:eaan2306. doi: 10.1126/scitranslmed.aan2306 29593104 PMC5918293

[91] Sokol H Pigneur B Watterlot L Lakhdari O Bermúdez-Humarán LG Gratadoux JJ . Faecalibacterium prausnitzii is an anti-inflammatory commensal bacterium identified by gut microbiota analysis of Crohn disease patients. Proc Natl Acad Sci USA. (2008) 105:16731–6. doi: 10.1073/pnas.0804812105 18936492 PMC2575488

[92] Guinane CM Cotter PD . Role of the gut microbiota in health and chronic gastrointestinal disease: Understanding a hidden metabolic organ. Therap Adv Gastroenterol. (2013) 6:295–308. doi: 10.1177/1756283x13482996 23814609 PMC3667473

[93] Wu HJ Ivanov II Darce J Hattori K Shima T Umesaki Y . Gut-residing segmented filamentous bacteria drive autoimmune arthritis via T helper 17 cells. Immunity. (2010) 32:815–27. doi: 10.1016/j.immuni.2010.06.001 20620945 PMC2904693

[94] Forkel M van Tol S Höög C Michaëlsson J Almer S Mjösberg J . Distinct alterations in the composition of mucosal innate lymphoid cells in newly diagnosed and established Crohn's disease and ulcerative colitis. J Crohns Colitis. (2019) 13:67–78. doi: 10.1093/ecco-jcc/jjy119 30496425

[95] Villarino AV Kanno Y Ferdinand JR O'Shea JJ . Mechanisms of Jak/Stat signaling in immunity and disease. J Immunol. (2015) 194:21–7. doi: 10.4049/jimmunol.1401867 25527793 PMC4524500

[96] Mangalam A Shahi SK Luckey D Karau M Marietta E Luo N . Human gut-derived commensal bacteria suppress Cns inflammatory and demyelinating disease. Cell Rep. (2017) 20:1269–77. doi: 10.1016/j.celrep.2017.07.031 28793252 PMC5763484

[97] Jiang L Shang M Yu S Liu Y Zhang H Zhou Y . A high-fiber diet synergizes with Prevotella copri and exacerbates rheumatoid arthritis. Cell Mol Immunol. (2022) 19:1414–24. doi: 10.1038/s41423-022-00934-6 36323929 PMC9709035

[98] Han EJ Ahn JS Chae YJ Chung HJ . Immunomodulatory roles of Faecalibacterium prausnitzii and Akkermansia muciniphila in autoimmune diseases: Mechanistic insights and therapeutic potential. Clin Rev Allergy Immunol. (2025) 68:77. doi: 10.1007/s12016-025-09093-8 40759811

[99] Laragione T Harris C Azizgolshani N Beeton C Bongers G Gulko PS . Magnesium increases numbers of Foxp3+ Treg cells and reduces arthritis severity and joint damage in an Il-10-dependent manner mediated by the intestinal microbiome. EBioMedicine. (2023) 92:104603. doi: 10.1016/j.ebiom.2023.104603 37201335 PMC10203746

[100] Sun Y Chen X Wang T Niu Y Cui M Li B . Siweixizangmaoru decoction attenuates collagen-induced arthritis via gut microbiota-dependent scfa restoration and immunomodulation. Phytomedicine. (2025) 148:157249. doi: 10.1016/j.phymed.2025.157249 40946662

[101] Liang H Li C Wang Y Hu Y Zhao X Luo N . Total flavonoids of Drynaria fortunei ameliorates rheumatoid arthritis by suppressing P. copri-mediated Th17 cell differentiation. J Ethnopharmacol. (2026) 358:120970. doi: 10.1016/j.jep.2025.120970 41317812

[102] Ma L Morel L . Loss of gut barrier integrity in lupus. Front Immunol. (2022) 13:919792. doi: 10.3389/fimmu.2022.919792 35795671 PMC9250981

[103] Kalayci FNC Ozen S . Possible role of dysbiosis of the gut microbiome in sle. Curr Rheumatol Rep. (2023) 25:247–58. doi: 10.1007/s11926-023-01115-8 37737528

[104] Botía-Sánchez M Galicia G Albaladejo-Marico L Toro-Domínguez D Morell M Marcos-Fernández R . Gut epithelial barrier dysfunction in lupus triggers a differential humoral response against gut commensals. Front Immunol. (2023) 14:1200769. doi: 10.3389/fimmu.2023.1200769 37346043 PMC10280985

[105] Nagi R Kumar SS Sheth M Deshpande A Khan J . Association between oral microbiome dysbiosis and Sjogren syndrome. A systematic review of clinical studies. Arch Oral Biol. (2025) 172:106167. doi: 10.1016/j.archoralbio.2024.106167 39798503

[106] Wang X Pang K Wang J Zhang B Liu Z Lu S . Microbiota dysbiosis in primary Sjögren's syndrome and the ameliorative effect of hydroxychloroquine. Cell Rep. (2022) 40:111352. doi: 10.1016/j.celrep.2022.111352 36103827

[107] Bao J Li L Zhang Y Wang M Chen F Ge S . Periodontitis may induce gut microbiota dysbiosis via salivary microbiota. Int J Oral Sci. (2022) 14:32. doi: 10.1038/s41368-022-00183-3 35732628 PMC9217941

[108] Arvidsson G Czarnewski P Johansson A Raine A Imgenberg-Kreuz J Nordlund J . Multimodal single-cell sequencing of B cells in primary Sjögren's syndrome. Arthritis Rheumatol. (2024) 76:255–67. doi: 10.1002/art.42683 37610265

[109] iMSMS Consortium . Gut microbiome of multiple sclerosis patients and paired household healthy controls reveal associations with disease risk and course. Cell. (2022) 185:3467–3486.e16. doi: 10.1016/j.cell.2022.08.021 36113426 PMC10143502

[110] Schwerdtfeger LA Montini F Lanser TB Ekwudo MN Zurawski J Tauhid S . Gut microbiota and metabolites are linked to disease progression in multiple sclerosis. Cell Rep Med. (2025) 6:102055. doi: 10.1016/j.xcrm.2025.102055 40185103 PMC12047500

[111] Tobin D Vige R Calder PC . Review: The nutritional management of multiple sclerosis with propionate. Front Immunol. (2021) 12:676016. doi: 10.3389/fimmu.2021.676016 34394076 PMC8355737

[112] Ahn JH da Silva Pedrosa M Lopez LR Tibbs TN Jeyachandran JN Vignieri EE . Intestinal E. coli-produced yersiniabactin promotes profibrotic macrophages in Crohn's disease. Cell Host Microbe. (2025) 33:71–88.e9. doi: 10.1016/j.chom.2024.11.012 39701098 PMC12488000

[113] Wang X He L Dong Y Qin X Zhang H Wang B . Mucosa-associated bacteria and metabolites in inflammatory bowel disease: From inside to insight. NPJ Biofilms Microb. (2026) 12:35. doi: 10.1038/s41522-025-00887-4 41617703 PMC12877147

[114] Dou Y Liu P Ding Z Zhou Y Jing H Ren Y . Orally administrable H(2) S-scavenging metal-organic framework prepared by co-flow microfluidics for comprehensive restoration of intestinal milieu. Adv Mater. (2023) 35:e2210047. doi: 10.1002/adma.202210047 36637449

[115] Kou R Guo Y Qin Z Xu X Liu Y Wei W . Systemic dysregulation of the gut microenvironment plays a pivotal role in the onset and progression of inflammatory bowel disease. Front Immunol. (2025) 16:1661386. doi: 10.3389/fimmu.2025.1661386 41080582 PMC12507907

[116] Zhang L Zhang YJ Chen J Huang XL Fang GS Yang LJ . The association of Hla-B27 and Klebsiella pneumoniae in ankylosing spondylitis: A systematic review. Microb Pathog. (2018) 117:49–54. doi: 10.1016/j.micpath.2018.02.020 29438717

[117] Asquith M Elewaut D Lin P Rosenbaum JT . The role of the gut and microbes in the pathogenesis of spondyloarthritis. Best Pract Res Clin Rheumatol. (2014) 28:687–702. doi: 10.1016/j.berh.2014.10.018 25488778 PMC4293151

[118] Berland M Meslier V Berreira Ibraim S Le Chatelier E Pons N Maziers N . Both disease activity and Hla-B27 status are associated with gut microbiome dysbiosis in spondyloarthritis patients. Arthritis Rheumatol. (2023) 75:41–52. doi: 10.1002/art.42289 35818337 PMC10099252

[119] Lou J Jiang Y Rao B Li A Ding S Yan H . Fecal microbiomes distinguish patients with autoimmune hepatitis from healthy individuals. Front Cell Infect Microbiol. (2020) 10:342. doi: 10.3389/fcimb.2020.00342 32850468 PMC7416601

[120] Mousa WK Chehadeh F Husband S . Microbial dysbiosis in the gut drives systemic autoimmune diseases. Front Immunol. (2022) 13:906258. doi: 10.3389/fimmu.2022.906258 36341463 PMC9632986

[121] Wei Y Li Y Yan L Sun C Miao Q Wang Q . Alterations of gut microbiome in autoimmune hepatitis. Gut. (2020) 69:569–77. doi: 10.1136/gutjnl-2018-317836 31201284

[122] Zhu Q Hou Q Huang S Ou Q Huo D Vázquez-Baeza Y . Compositional and genetic alterations in Graves' disease gut microbiome reveal specific diagnostic biomarkers. ISME J. (2021) 15:3399–411. doi: 10.1038/s41396-021-01016-7 34079079 PMC8528855

[123] Benhadou F Mintoff D Schnebert B Thio HB . Psoriasis and microbiota: A systematic review. Diseases. (2018) 6:47. doi: 10.3390/diseases6020047 29865237 PMC6023392

[124] Scher JU Ubeda C Artacho A Attur M Isaac S Reddy SM . Decreased bacterial diversity characterizes the altered gut microbiota in patients with psoriatic arthritis, resembling dysbiosis in inflammatory bowel disease. Arthritis Rheumatol. (2015) 67:128–39. doi: 10.1002/art.38892 25319745 PMC4280348

